# Exploring the effects of gender and sexual orientation on disordered eating: an EFA to CFA study of the Eating Disorder Examination Questionnaire

**DOI:** 10.1186/s40337-023-00821-z

**Published:** 2023-06-22

**Authors:** Ruth Knight, Catherine Preston

**Affiliations:** 1 Department of Psychology, York St John University, Lord Mayors Walk, York, Y031 7EX UK; 2 University of York, York, UK

**Keywords:** Disordered eating, Gender, Sexual orientation, Muscularity, Marginalised communities

## Abstract

Several problems limit our understanding of the ways that gender and sexual orientation influence disordered eating. These include the reliance on measures that have been developed and validated in samples of cisgender heterosexual women, and the lack of confirmed measurement invariance that allows us to meaningfully compare these experiences between groups. This study was an EFA to CFA exploration of the Eating Disorder Examination Questionnaire in a group of heterosexual, bisexual, gay, and lesbian men and women. In total 1638 participants were recruited via adverts in traditional and social media to complete an online survey. A 14-item, three-factor model of the EDE-Q was confirmed as best fitting the data and measurement invariance between groups was ascertained. Sexual orientation influenced disordered eating and muscularity-related thoughts and behaviours in men but not women. Heterosexual men reported more muscularity-related concerns and behaviours, whereas gay men showed more thinness-related concerns and behaviours. Bisexual participants showed a different pattern, highlighting the importance of treating this group individually and not collating all non-heterosexual participants together. Small but significant effects of sexual orientation and gender have an impact on the kinds of disordered eating thoughts and behaviours one might experience, and could influence prevention and treatment. Clinicians may be able to provide more effective and tailored interventions by taking into account gender and sexual orientation in sensitive ways.

## Abstract (plain language summary)

Our idea of what an eating disorder looks like is tied to the kind of participant that is usually included in research. However, a wide range of people struggle with their body image. It can be difficult to measure what kinds of experiences these individuals have as the questionnaires typically used were not validated in marginalised groups. This study aims to see if a commonly used questionnaire is useful in men and women identifying as gay, lesbian, bisexual, and heterosexual and if there are differences in the kinds of disordered eating thoughts and behaviours that these groups experience. A version of the questionnaire with three factors fits this data well. Sexual orientation does seem to influence the rates of disordered eating in men but not women. Heterosexual men experience more behaviours that might be related to muscularity, whereas gay men show more concerns regarding body shape and leanness. Bisexual men show lower levels of disordered eating. We suggest that considering gender and sexual orientation when treating disordered eating may help clinicians to tailor interventions. Our idea of what an eating disorder looks like is a narrow view of a much broader picture.

## Introduction

Eating disorders (ED) are serious psychiatric conditions with notoriously high mortality [65] and relapse [20] rates. Historically ED are associated with young, slim, heterosexual women [54] and research investigating ED aetiology in other groups, including men and sexual minorities, is lacking despite men making up at least one in four diagnosed cases and sexual minorities exhibiting poorer mental health in general [10, 17]. Indeed, much of the prior research excludes men and sexual minorities, considering them atypical despite EDs being reported in men for as long as they have been reported in women and some previous studies demonstrating that sexual minority participants show higher rates of these behaviours [28, 53]. The original measures have either not been validated in these communities, or when validation has been explored research predominantly focuses on men and women separately, meaning that comparisons between groups cannot be made.

Although clinical EDs are thought to present with similar symptoms in men and women [21], there are significant differences between genders in community samples [6]. For example, men generally score lower than women on attitudinal aspects of ED symptomatology [6, 42]. This may reflect a qualitative difference in the way that men and women interpret assessment questions rather than a quantitative difference in rates of these experiences [6, 40, 66]. This is not surprising given that measures are typically developed in heterosexual cisgender women, and the majority have not been adapted for current DSM ED diagnoses [6, 52, 66]. Therefore, it is difficult to ascertain whether differences between men and women reflect differences in rates of disordered eating, or whether they could more accurately be said to reflect differences in the kinds of disordered eating thoughts and behaviours that an individual might experience. Understanding and addressing body-related disorders in different groups requires the ability to correctly identify their presence, which calls for more accurate, validated measures and nuanced accounts of rates of different kinds of disordered eating thoughts and behaviours across different groups [40, 74]. Being able to meaningfully explore the differences between gender and sexual orientation groups in terms of disordered eating will allow us to test theories around why differences might exist, as well as helping us to uncover the ways that gender identity and sexual orientation influence the kinds of symptoms that an individual might experience. This facilitates the development of appropriate preventative and treatment strategies, instead of applying a one size fits all approach.

Previous meta-analysis concluded that differences in disordered eating thoughts and behaviours between women of different sexual orientations were small enough that they were not meaningful [49]. Results from more recent meta-analyses also indicate that there is a low likelihood of heterosexual and lesbian women experiencing tangible differences in body image disturbance [13, 29]. However, many of the measures included in these analyses have not been validated in different samples, such as in men and women from sexual minorities.

Previous findings focused predominantly on general body dissatisfaction and did not consider aspects that may be more strongly influenced by modern social media-driven body ideals, such as drive for muscularity (linked to fitspiration trends) and related restrictive eating behaviours. Meta-analyses suggest that gay men experience more body dissatisfaction than heterosexual men, but less than women who identified as either heterosexual or lesbian (although effect sizes for these differences are small) [13]. Recent research validated the EDE-Q for use in men of different sexual orientation groups and indicated that heterosexual men show higher rates of restriction-related behaviours, which may be linked to muscularity, whereas gay men show more thoughts and behaviours related to body shape and weight [40]. Other research explored rates of these behaviours amongst sexual minorities, but did not directly compare these individual groups with heterosexual samples [26].

Only recently has research begun to include bisexual participants independently, previously they were excluded or combined with gay and lesbian participants into a non-heterosexual group [4, 12, 22, 23]. A recent study indicated that bisexual men show less disordered eating thoughts and behaviours than gay men, however the study did not include female participants meaning results are limited to men [40]. Further exploration of how bisexual participants experience disordered eating may enable a fuller understanding of how sexuality itself influences these symptoms. Currently the majority of research focuses on gay or heterosexual peoples’ experiences, so the inclusion of bisexual participants across genders would allow a more detailed picture of the link between sexuality and disordered eating, given that bisexual participants may experience different pressures to other groups. Crucially, there is very little disordered eating research available that explores the effect of both gender and sexual orientation, with the majority of research focusing on one or the other in isolation.

A key symptom that is not captured in traditional ED assessments, but thought to be important for evaluating male attractiveness, and increasingly women's attractiveness, is muscularity [16, 53]. The internalization of muscularity alongside the thin-ideal is associated with body dissatisfaction and eating concerns in men [39]. Moreover, muscle dysmorphia (misestimation of one's own muscularity) is considered the male equivalent of body size overestimation, which is frequently linked to disordered eating in women [3, 35, 64]. Some studies suggest that gay men have a stronger drive for muscularity than heterosexual men [76], whilst others suggest that sexuality does not influence drive for muscularity in men [55]. There is evidence that sexual minority men tend to place more importance on muscularity than thinness [44, 75, 76]. This may link to the pressure on sexual minority men to appear masculine, a trait that is often associated with muscularity [36]. This, combined with pressure to be physically attractive, may result in a pressure to conform to a lean and muscular body ideal [74]. However research is limited, and the way in which sexuality relates to drive for muscularity and ED symptoms in men is not yet clear.

There is significantly less research available around the drive for muscularity in women, despite increasing exposure to fitspiration content that promotes lean muscularity as attractive in for this group [32]. There is also very little empirical research attending to the effects of both gender and sexual orientation on these behaviours, with studies again focussing on only one or the other. Some researchers suggest that muscularity for men is the equivalent of thinness for women (hence the term ‘bigorexia’) [24]. However, very few studies have directly compared drive for muscularity related thoughts and behaviours across men and women, alongside how this relates to sexual orientation.

Taking this into consideration, we suggest that current work regarding the relationships between sexual orientation, gender identity and disordered eating suffers from at least three problems that hinder our understanding. Firstly, finding measures that are appropriate for the wide range of people who experience disordered eating. Secondly, potential influences of gender on the kinds of thoughts and behaviours that people experience, as well as the rates of these. Thirdly, the influence of sexual orientation on the rates and kinds of disordered eating experiences and how this interacts with gender. Therefore, we explored whether identifying as gay, bisexual, or heterosexual impacted prevalence of disordered eating symptoms and drive for muscularity in a community sample of men and women as examined through the Eating Disorder Examination Questionnaire (EDE-Q) and the Drive for Muscularity scale (DMS). This extends previous work that established measurement invariance in the EDE-Q in men, and allowed comparison across sexual orientation groups. The majority of previous research has focussed on either muscularity *or* ED attitudes, sexual orientation *or* gender, and often has not considered bisexual participants as a group in their own right. In addition to this, modern definitions of the ideal body type being both lean and muscular may suggest an important link between disordered eating symptoms and drive for muscularity in both men and women.

Firstly, we examined the appropriateness of the standard EDE-Q as a measure of disordered eating by testing model fit in a large community sample and used the best fit measure for all subsequent analyses. Based on the numerous previous studies that find that the original four-factor structure is not a good fit, as well as changes in society and understandings of what constitutes an ED since the measure’s conception, we predicted that the original four-factor structure would be a poor fit. We then examined, through measurement invariance analysis, whether there was evidence that the EDE-Q is measuring equivalent constructs for participants of different sexual orientations and genders. We considered rates of attitudes and behaviours relating to disordered eating and drive for muscularity in gay, lesbian, bisexual, and heterosexual participants.

Given that research indicates muscularity is increasingly relevant to disordered eating [48, 52], we include the Drive for Muscularity Scale in this study. Traditional eating disorder measures like the EDE-Q do not capture this, so we included the DMS to give a more comprehensive picture of the experiences each group has related to body satisfaction. This measures the importance of muscularity to an individual in terms of both attitudes and behaviours, in a similar way to the EDE-Q measuring more traditional disordered eating thoughts and behaviours, making them especially useful measures when taken together. The DMS has already been validated for use in sexual minorities alongside heterosexual participants, and we used this model in this study [37]. The EDE-Q has previously been validated for use in men of different sexual orientations [40], thus we used the measure here to extend these findings and determine the most appropriate structure for both men and women of different sexual orientations.

## Methods

### Participants

In total 2975 participants took part in the study, and 1737 of these completed both of the measures in question. There were insufficient numbers of participants who were agender, non-binary, demi-gender, or asexual to include them in analysis. In total there were 1638 participants identifying as either male (1047) or female (591) and heterosexual (men = 525, women = 152), gay (276) or lesbian (159), or bisexual (men = 246, women = 280). Gender was operationalized as the gender that the participant identified as; participants could choose from a set of labels or use a free text box to describe their gender. Sexual orientation was operationalized as the sexual identity that participants declared. Participants were on average 27 years old (SD = 8.44). 80% of participants identified as White, 3.8% as Hispanic/Latino, 5% as Black, and 5.48% as Asian. 4.52% identified as other (15 participants identified as Mixed Race, 1 as Native American, and one as Romany), and 0.24% chose not to answer. Much of the sample (44.32%) had completed a Bachelor’s degree or higher. However, the participant group includes both students and non-students.

### Measures

#### Eating Disorder Examination Questionnaire (EDE-Q) 6.0

The EDE-Q is a 28-item self-report questionnaire that assesses eating disorder symptoms [18, 19]. Participants rate items on a 7-point Likert scale, with higher scores indicating higher eating disorder psychopathology. Six items relate to the frequency of eating disorder attitudes and behaviours in the past 28 days, and do not contribute to subscale or global scores but provide information on core eating disorder behaviours such as laxative use and self-induced vomiting. These were not examined in this analysis. Research has established acceptable levels of internal consistency and reliability for global and subscale scores in men and women [2, 31, 42, 58, 62].

#### Drive for muscularity scale (DMS)

The DMS is a 15-item self-report questionnaire that assesses how important being muscular is to participants, and how they act to develop their muscularity. Participants answer each item on a 5-point Likert scale anchored by ‘Always’ and ‘Never’. The scale uses reverse scoring on all items. Higher scores indicate higher drive for muscularity, providing muscularity-driven behaviours and muscularity-oriented attitudes scores individually, as well as an overall score. The item has shown acceptable reliability and validity in male samples [46]. Research has established the optimal factor structure in sexual minority men and women, as well as measurement invariance across genders [38] thus, we did not assess the factor structure or invariance across sexual orientation groups in this study. We used the 14-item two-factor model that has been established as the best fit for sexual minority men and women [38].

### Procedure

Participants were invited to take part in an online questionnaire examining feelings toward the body and sexual orientation through adverts on social media, in local community groups, and in Attitude magazine (print and digital). Participants followed a link to an online questionnaire delivered via Qualtrics (Qualtrics, Provo, UT). The questionnaire included demographic information (age, gender, sexuality, and ethnicity), followed each time by the EDE-Q and then the DMS. The survey took approximately 20 min to complete.

### Data analysis

To explore the factor structure of the EDE-Q, we used an Exploratory Factor Analysis (EFA) to Confirmatory Factor Analysis (CFA) approach [70]. We split the overall sample using a computer-generated random number to get equal data points from each group based on sexuality and gender. We used this split sample for the EFA and retained the remaining data for the CFA. There were no significant differences in basic demographics (age, ethnicity, gender) between the two samples.

We conducted an EFA using the lavaan, psych, and GPArotation packages in R. We assessed the suitability of this data for factor analysis using Kaiser–Meyer–Olkin (KMO) measure of sampling adequacy, with a value of 0.80 being ideal and 0.60 being adequate [34] and Bartlett’s test of sphericity, which should be significant. We used maximum likelihood estimation with an oblique oblimin rotation as research suggests that factors of the EDE-Q are correlated. The EFA included both men and women as the aim of the study was to derive a factor structure suitable for mixed samples.

To determine how many factors to extract, we used parallel analysis alongside examination of fit indices [71]. We retained items based on the recommendations that items with loadings > 0.40 with low inter-item correlations should be kept whilst bearing in mind the recommendation that factors with fewer than three items should be excluded, alongside those that explain less than 5% of the variance [11, 25, 72, 73]. A cut-off of loadings of 0.40 was used for retaining factors [67]. We followed criteria set out as best practice to assess the fit of the model [33]. This states that Standardized Root Mean Residual (SRMR) should be less than 0.08, and Comparative Fit Index (CFI) and Tucker-Lewis Index (TLI) should be close to 0.95. A root mean square error of approximation (RMSEA) value of < 0.06 indicates good fit and 0.07–0.08 shows adequate fit. The above values should not be used in a rigid way given the other factors that may influence these values [70].

We used these values to assess model fit in the CFA. The information provided by modification indices can influence the fit of the model. Modification indices give an estimate of the increase in chi-squared for a fixed parameter if it were to be freed [57]. This is particularly relevant for scales in which items might be correlated, as is the case with the EDE-Q. However, one should also practice caution when using this approach, as it is data-driven as opposed to theoretical [57]. When modification indices are used, a cut -off of at least 3.84 is recommended, although more conservative accounts suggest a cut-off of at least 5.00 [5]. We used a cut-off of at least 3.84.

We conducted measurement invariance analysis of the EDE-Q between the gender and sexuality groups using a multigroup CFA approach. We examined this at the configural level (whether the number of latent variables and loadings are similar across groups) and then at the metric level (assessing whether the magnitude of the loadings are similar across groups). At the scalar level we assessed whether item loadings and intercepts are similar across groups. Strict invariance suggests the residuals are similar across groups [61]. A difference in CFI of less than 0.01 indicated metric invariance. Scalar invariance is supported by a difference in CFI of less than 0.01 as well as a difference in RMSEA of less than 0.015 or a difference in SRMR of less than 0.030 [7]. Some suggest that the difference of less than 0.01 in CFI is sufficient to indicate scalar invariance [8]. We use the difference in CFI combined with differences in RMSEA or SRMR. We used Omega (McDonald’s ω) values alongside Cronbach’s alpha to establish reliability of scores. Having established at least scalar invariance, we compared EDE-Q scores between gender and sexual orientation groups.

## Results

We first tested the fit of the traditional four-factor model on the first split half of the data, which included 420 participants, with 70 participants from each group. Results indicated that this was not a good fit for the data with all fit indices falling well below acceptable cut-off scores, even following modification indexing [chi squared (183) = 2407.554, *p* < 0.001, CFI = 0.791, TLI = 0.762, RMSEA = 0.12, SRMR = 0.097]. We proceeded to identify a better-fitting model using an EFA.

### EFA

The half of the data that the EFA was carried out on consisted of 420 participants (70 participants from each group.) This was a sufficient sample based on recommendations in the literature, reaching at least 15 participants per item [56, 73]. Bartlett’s test of sphericity, chi squared (120) = 5564.052*, p* < 0.001, and the KMO measure of sampling adequacy, KMO = 0.92, indicated that the EDE-Q has sufficient common variance for factor analysis. EFA and parallel analysis indicated that three factors should be extracted. The factors explained 69% of the common variance. The fit indices for the model are chi squared (52) = 4550.13, *p* < 0.001, CFI = 0.971, TLI = 0.948, RMSEA = 0.079 (90% CI = 0.0.067, 0.092), SRMR = 0.03. This indicated that the three-factor model is an adequate to good fit, with CFI and SRMR showing good fit, TLI close to good fit, and RMSEA showing adequate fit. Considering advice cautioning against dismissing models for not adhering to strict cut-offs [50] and that all indices indicated at least an adequate fit, we continued with this model. Factor loadings, reported in Table 1, indicated that 14 items should be retained (those with split loadings were removed from analysis: items 10, 12, 20, and 24; alongside those with factor loadings less than 0.40: items 2 and 6). The factors retained were Preoccupation and Eating Concern, Shape and Weight Concern, and Restriction. This differs from the originally proposed four-factor model in that here Shape and Weight Concern are combined.

**Table 1:** A table showing EFA results

Item	Shape and weight concern	Preoccupation and eating concern	Restriction
Q1—deliberately trying to limit food to influence shape or weight	0.06	− 0.03	**0.85**
Q2—going for long periods without eating to influence shape or weight	0.1	0.15	0.25
Q3—tried to exclude foods that you like to influence shape or weight	0.02	0.02	**0.81**
Q4—tried to follow definite rules to influence shape or weight	− 0.07	0.05	**0.75**
Q5—desire to have empty stomach to influence shape or weight	0.13	**0.41**	0.2
Q6—desire for a flat stomach	0.26	0.11	0.28
Q7—thinking about food/eating/calories made it difficult to concentrate	− 0.07	**0.93**	− 0.01
Q8—thinking about shape or weight made it difficult to concentrate	0.02	**0.95**	− 0.01
Q9—fear of losing control over eating	0.13	**0.69**	0.06
Q10—fear of gaining weight	**0.48**	**0.41**	0.02
Q11—felt fat	**0.72**	0.06	0.14
Q19—eaten in secret	0.12	**0.46**	− 0.03
Q20—felt guilt over eating	**0.45**	**0.41**	0.02
Q22—weight influenced how feel about self	**0.62**	0.2	0.14
Q23—shape influenced how feel about self	**0.38**	0.16	0.13
Q24—upset if asked to weigh self once a week	**0.47**	0.35	− 0.19
Q25—dissatisfied with weight	**0.85**	0	0.07
Q26—dissatisfied with shape	**0.90**	− 0.05	0.09
Q27—discomfort at seeing body	**0.96**	− 0.01	− 0.1
Q28—discomfort at others seeing shape or figure	**0.89**	0.03	− 0.1

Bold type indicates retained factor

### CFA

Monte Carlo simulation indicated that 450 would be a sufficient sample size for CFA. We conducted CFA on the 14-item model derived from the initial EFA with the second half of the sample, results for which can be found in Table 2. This sample was made up of 170 bisexual men, 180 heterosexual men, 202 gay men, 206 bisexual women, 76 heterosexual women, and 85 lesbian women.

**Table 2:** A table showing CFA results for the 14 item model, with and without allowing items to correlate

Model	Sample	Chi squared	CFI	TLI	RMSEA	SRMR
14 item	Combined	Chi squared (74) = 551.910, *p* < .001	0.944	0.931	0.085 (0.078–0.091)	0.051
14 item (with MI)	Combined	Chi squared (58) = 202.147, *p* < .001	0.983	0.974	0.052 (0.045–0.060)	0.03

As EDE-Q items are often related to one another, and in fact sometimes items in the same factor are identical aside from a change in the word “weight” or “shape”, we allowed items to correlate based on Byrne et al. [5], and used a conservative modification indices cut-off of ≥ 5.00. We tested this on the second split half of the sample (N = 904). Model fit indices were close to a good fit initially and when modification indices were used to free relevant parameters the model showed good fit on all indices. Based on these results we used the 14-item model in future analysis (see Table 2 for further information.)

### Measurement invariance

It is suggested that between-groups comparisons of mean scores should not be made in the absence of scalar invariance [70]. We used the suggested change in CFI as being less than 0.01 as sufficient for metric invariance and change in CFI as less than 0.01 plus change in RMSEA of less than 0.015 or a difference in SRMR of less than 0.030 to ascertain this [7]. The 14-item model reached scalar invariance between gender groups [change in CFI = 0.007, change in RMSEA = 0.002 and change in SRMR = 0.003 (see Table 3)]. The model reached at least scalar invariance between sexuality groups. Based on this we conducted between-group analysis using the 14-item model.

**Table 3:** A table showing measurement invariance analysis between gender groups

	Chi squared	CFI	TLI	RMSEA (95% CIs)	SRMR
Configural	Chi squared (148) = 645.801, *p* < .001	0.943	0.930	0.086 (0.080–0.093)	0.054
Metric	Chi squared (159) = 658.685, *p* < .001	0.942	0.934	0.083 (0.077–0.090)	0.056
Scalar	Chi squared (170) = 730.975, *p* < .001	0.935	0.931	0.085 (0.079–0.092)	0.059
Strict	Chi squared (184) = 828.886, *p* < .001	0.926	0.927	0.088 (0.082–0.094)	0.060

### Scale reliability

For the CFA sample, overall scale reliability was demonstrated through Cronbach’s alpha of 0.913 and McDonald’s ω of 0.920, suggesting excellent reliability. We also calculated the reliability of each factor individually. For Shape and Weight Concern, Cronbach’s alpha was 0.935 and McDonald’s ω was 0.936, indicating excellent reliability. For Preoccupation and Eating Concern, Cronbach’s alpha was 0.838 and McDonald’s ω was 0.847, indicating good reliability. For Restriction, Cronbach’s alpha was 0.786 and McDonald’s ω was 0.786, indicating acceptable reliability.

For the DMS, Cronbach’s alpha was 0.934 and McDonald’s ω was 0.935, indicating excellent reliability. For the DMS Attitudes factor, Cronbach’s alpha was 0.899 and McDonald’s ω was 0.902, indicating good to excellent reliability. For the DMS Behaviours factor, Cronbach’s alpha was 0.916 and McDonald’s ω was 0.920. These values indicate that reliability was at least sufficient for both measures.

### Comparison of scores

Further analysis for the EDE-Q was conducted on a random subset of participants (selected from across both EFA and CFA samples), as groups were not equal in size in the original sample, which was generated using a random seed. In this subset, N = 200 bisexual women, N = 159 lesbian women, N = 152 heterosexual women, N = 200 bisexual men, N = 200 gay men, and N = 200 heterosexual men. Not all the participants completed both the EDE-Q and the DMS, so for the DMS sample there were N = 269 bisexual women, N = 266 lesbian women, N = 251 heterosexual women, N = 281 bisexual men, N = 154 gay men, and N = 172 heterosexual men. Cronbach’s alpha for this sample was 0.915 and Mcdonald’s ω was 0.920 demonstrating good internal consistency. Data met the assumptions for an ANOVA. A one-way ANOVA found that age was significantly different across sexuality groups for the EDE-Q [F(5,1108) = 106.86, *p* < 0.001, ηp^2^ = 0.325] and for the DMS [F(5, 1387) = 87.29, *p* < 0.001, ηp^2 =^ 0.239]. Due to this, age was controlled for in all subsequent analysis.

A 2 × 3 MANCOVA on the EDE-Q scales (Shape and Weight Concern, Preoccupation and Eating Concern, Restriction) with the factors gender (male, female) and sexuality (heterosexual, gay/lesbian, bisexual) revealed a significant main effect of sexuality [F(6,2210) = 10.96, Wilks lambda = 0.94, *p* < 0.001] and gender [F(3,1105) = 19.59, Wilks lambda = 0.96, *p* < 0.001]. There was also a significant interaction between gender and sexuality [F(6,2210) = 15.49, Wilks lambda = 0.92, *p* < 0.001] and a significant effect of age [F(3,1105) = 6.38, Wilks lambda = 0.983, *p* < 0.001]. To follow up the MANCOVA results we conducted univariate analysis on each of the subscales. Means and SDs for each group can be found in the Figures.

For the Shape and Weight Concern factor, Cronbach’s alpha was 0.938 and Mcdonald’s ω was 0.939 demonstrating good internal consistency. A 2 × 3 ANCOVA was conducted on Shape and Weight Concern scores with the factors gender (male, female) and sexuality (heterosexual, gay/lesbian, bisexual) and age as a covariate. There was no significant main effect of gender [F(1, 1107) = 1.62, *p* = 0.204, ηp^2^ = 0.001]. There was a significant main effect of sexuality [F(2, 1107) = 7.305, *p* < 0.001, ηp^2^ = 0.013]. There was also a significant gender*sexuality interaction [F(2, 1107) = 25.24, *p* < 0.001, ηp^2^ = 0.044], but no significant effect of age [F(1, 1107) = 0.9, *p* = 0.343, ηp^2^ = 0.001].

To follow up on the significant main effect of sexuality on Shape and Weight Concern, we conducted Bonferroni corrected pairwise comparisons (the Bonferroni critical *p-*value is 0.025). Gay/lesbian participants scored significantly higher than heterosexual participants [t(711) = 5.21, *p* < 0.001, d = 0.39]. However, there was no significant difference between bisexual and heterosexual participants [t(742) = 0.556, *p* = 0.578, d = 0.041].

**Fig. 1:**
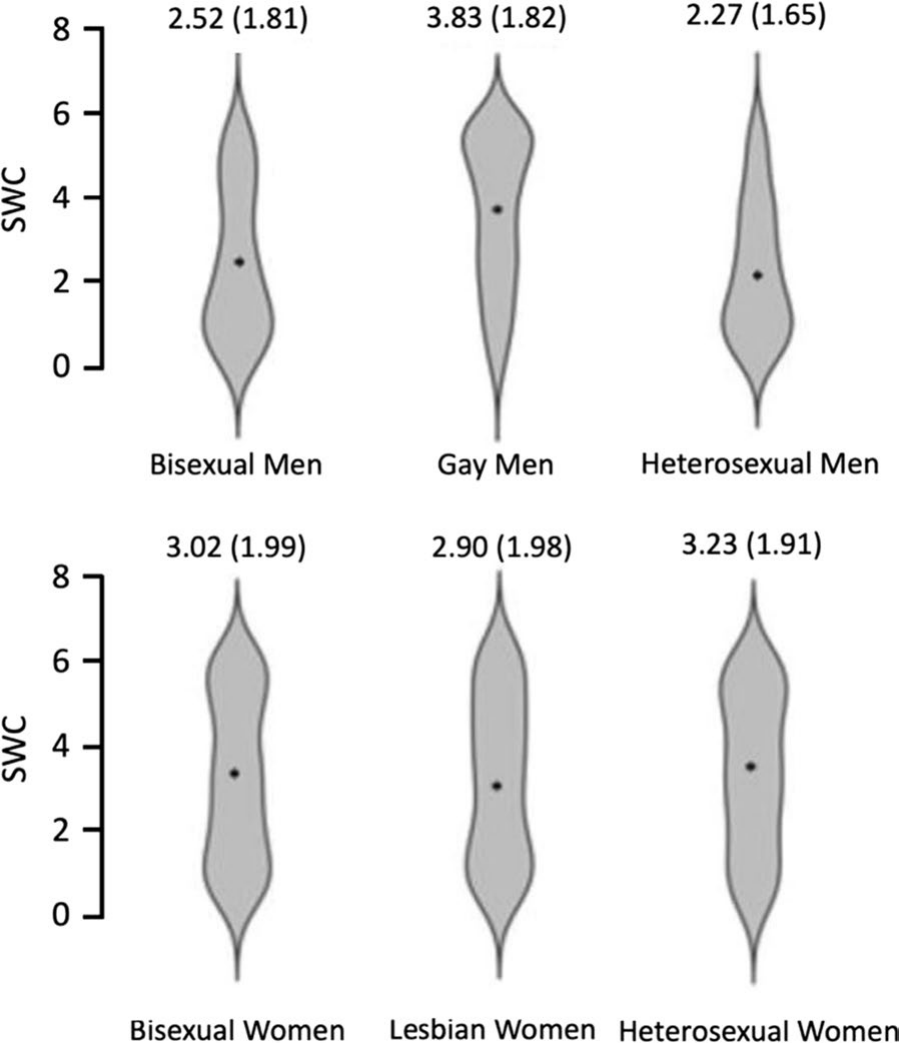
Two violin plots showing the small significant gender*sexuality interaction in Shape and Weight concern scores, such that there are no differences in scores amongst women, but gay men show higher scores than heterosexual men and women, and lesbian women. *Note* Means and SDs (in brackets) are displayed in the figure

To follow up on the significant gender*sexuality interaction, we conducted further Bonferroni corrected pairwise comparisons (the Bonferroni critical *p-*value is 0.0083). Gay men scored significantly higher on Shape and Weight Concern than heterosexual men [t(400) = 8.96, *p* < 0.001, d = 0.895]. However, there was no significant difference between bisexual and heterosexual men [t(390) = 1.44, *p* = 0.151, d = 0.145]. There was no significant difference in Shape and Weight Concern between bisexual and heterosexual women [t(350) =  − 0.976, *p* = 0.330, d = − 0.105] or between lesbian and heterosexual women [t(309) =  − 1.45, *p* = 0.147, d = − 0.165]. Gay men scored significantly higher on Shape and Weight Concern compared to both lesbian [t(368) = − 4.65, *p* < 0.001, d = − 0.498] and heterosexual women [t(361) = − 3.04, *p* = 0.003, d = − 0.324] (see Fig. 1).

For Preoccupation and Eating Concern, Cronbach’s alpha was 0.839 and Mcdonald’s ω was 0.847 demonstrating good internal consistency. A 2 × 3 ANCOVA was conducted on Preoccupation and Eating Concern scores with the factors gender (male, female) and sexuality (heterosexual, gay/lesbian, bisexual) and the covariate age. There was no significant main effect of gender [F(1, 1107) = 2.99, *p* = 0.084, ηp^2^ = 0.003]. There was a significant main effect of sexuality [F(2, 1107) = 3.77, *p* = 0.023, ηp^2^ = 0.007]. There was a significant gender*sexuality interaction [F(2, 1107) = 11.67, *p* < 0.001, ηp^2^ = 0.021] and no significant effect of age [F(1, 1107) = 1.63, *p* = 0.20, ηp^2^ = 0.001].

**Fig. 2:**
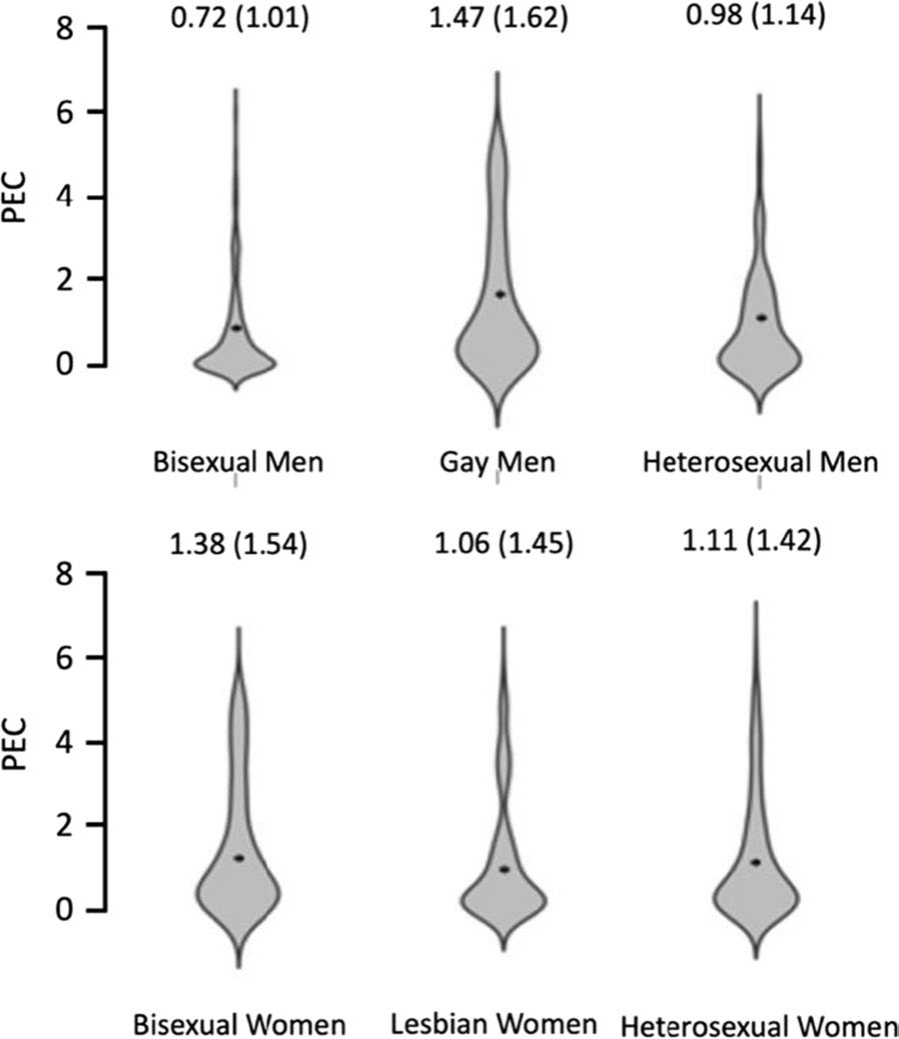
Two violin plots showing the small significant gender*sexuality interaction in Preoccupation and Eating Concern scores, such that there were no differences amongst women, but gay men score higher than heterosexual and bisexual men, but not differently from heterosexual and lesbian women. *Note* Means and SDs (in brackets) for each group are shown in the figure

To follow up on the significant main effect of sexuality, we conducted Bonferroni corrected pairwise comparisons (the Bonferroni critical *p-*value is 0.025). Gay/lesbian participants scored higher than heterosexual participants [(t(711) = 2.39, *p* = 0.017, d = 0.180]. There was no significant difference in scores between bisexual and heterosexual participants [t(742) = 0.112, *p* = 0.910, d = 0.008]. These results can be found in Fig. 2.

To follow up on the significant gender*sexuality interaction, we conducted further Bonferroni corrected pairwise comparisons (the Bonferroni critical *p-*value is 0.0083). We found that gay men had significantly higher scores than heterosexual men [t(400) = 3.46, *p* < 0.001, d = 0.345]. However, there were no significant differences between heterosexual and bisexual men [t(390) = − 2.29, *p* 0.022, d = − 0.232]. There were no significant differences between bisexual and lesbian women [t(350) = 1.68, *p* = 0.095, d = 0.180] or heterosexual and lesbian women [t(309) = − 0.285, *p* = 0.776, d = − 0.032] on this subscale. We did not find significant differences in Preoccupation and Eating Concern scores between gay men and heterosexual women [t(361) = − 2.22, *p* = 0.027, d = − 0.236] or between gay men and lesbian women [t(368) = − 2.52, *p* = 0.012, d = − 0.265].

For the Restriction subscale, Cronbach’s alpha was 1.00 and Mcdonald’s ω was 0.999 demonstrating good internal consistency. A 2 × 3 ANCOVA was conducted on Restriction scores with the factors gender (male, female) and sexuality (heterosexual, gay/lesbian, bisexual). There was a significant main effect of gender [F(1, 1107) = 35.12, *p* < 0.001, ηp^2^ = 0.031, such that men (M = 2.94, SD = 2.11) scored significantly higher than women (M = 2.22, SD = 2.02). There was a significant main effect of sexuality F(2, 1107) = 8.14, *p* < 0.001, ηp^2^ = 0.014]. There was also a significant gender*sexuality interaction [F(2, 1107) = 9.24, *p* < 0.001, ηp^2^ = 0.016] and a significant effect of age [F(1, 1107) = 6.14, *p* = 0.013, ηp^2^ = 0.006]. However, the effect size of the effect of age was small, and not supported by a follow-up correlation (r = 0.03, *p* = 0.307).

To follow up on the significant main effect of sexuality, we conducted Bonferroni corrected pairwise comparisons (the Bonferroni critical *p-*value is 0.025). There were no significant differences found in Restriction scores between gay/lesbian and heterosexual participants [t(711) = − 1.07, *p* = 0.284, d = − 0.080]. Heterosexual participants scored significantly higher on the Restriction subscale than bisexual participants [t(742) = − 4.47, *p* < 0.001, d = − 0.329].

**Fig. 3:**
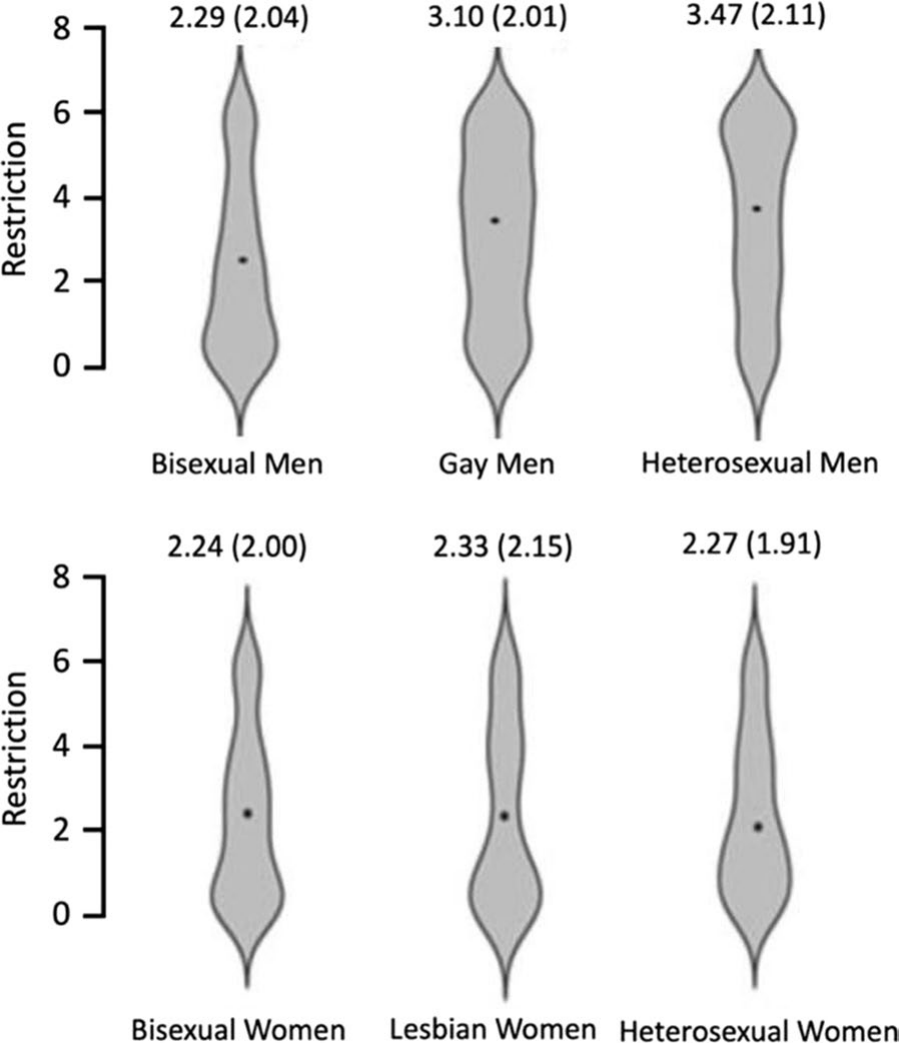
Two violin plots showing the small significant gender*sexuality interaction in Restriction scores, such that there were no differences between women, but gay men scored significantly higher than heterosexual and lesbian women. Heterosexual men scored higher than gay men. *Note* Means and SDs (in brackets) for each group are shown in the figure

To follow up on the significant gender*sexuality interaction, we conducted further Bonferroni corrected pairwise comparisons (the Bonferroni critical *p-*value is 0.0083). There were no significant differences between gay and heterosexual men [t(400) = − 1.80, *p* = 0.073, d = − 0.180]. Heterosexual men scored significantly higher than bisexual men [t(390) =  − 5.64, *p* < 0.001, d = − 0.570]. Neither bisexual [t(350) = − 0.165, *p* = 0.869, d = − 0.018] nor lesbian women [t(350) = − 0.165, *p* = 0.869, d = − 0.018] scored significantly differently from heterosexual women on Restriction scores. Gay men scored significantly higher than heterosexual women [t(361) = − 3.95, *p* < 0.001, d = − 0.420] and lesbian women [t(368) = − 3.53, *p* < 0.001, d = − 0.371]. However, given the pattern of results described above, these significant differences may reflect a main effect of gender with men scoring higher than women (Fig. 3).

### Drive for muscularity

For DMS, Cronbach’s alpha was 0.939 and Mcdonald’s ω was 0.940, indicating excellent reliability. For DMS Behaviours, Cronbach’s alpha was 0.905 and Mcdonald’s ω was 0.907, indicating excellent reliability. For DMS Attitudes, Cronbach’s alpha was 0.918 and Mcdonald’s ω was 0.922, indicating excellent reliability.

A 2 × 3 ANCOVA was conducted on DMS scores with the factors gender (male, female) and sexuality (heterosexual, gay/lesbian, bisexual) and the covariate of age. There was a significant main effect of gender [F(1, 1386) = 417.16, *p* < 0.001, ηp^2^ = 0.254] such that men scored significantly higher than women. There was a significant main effect of sexuality [F(2, 1386) = 28.69, *p* < 0.001, ηp^2^ = 0.040]. There was also a significant gender*sexuality interaction [F(2, 1386) = 40.44, *p* < 0.001, ηp^2^ = 0.055] and a significant effect of the covariant age [F(1, 1386) = 40.88, *p* < 0.001, ηp^2^ = 0.040]. A follow up correlation (r = − 0.19, *p* < 0.001) suggests that as age increases, drive for muscularity decreases.

To follow up on the significant main effect of sexuality, we conducted Bonferroni corrected pairwise comparisons (the Bonferroni critical *p-*value is 0.025). Heterosexual participants scored significantly higher on DMS than gay/lesbian participants [t(841) = − 5.14, *p* < 0.001, d = − 0.354] and bisexual participants [t(971) = − 8.87, *p* < 0.001, d = − 0.574].

**Fig. 4:**
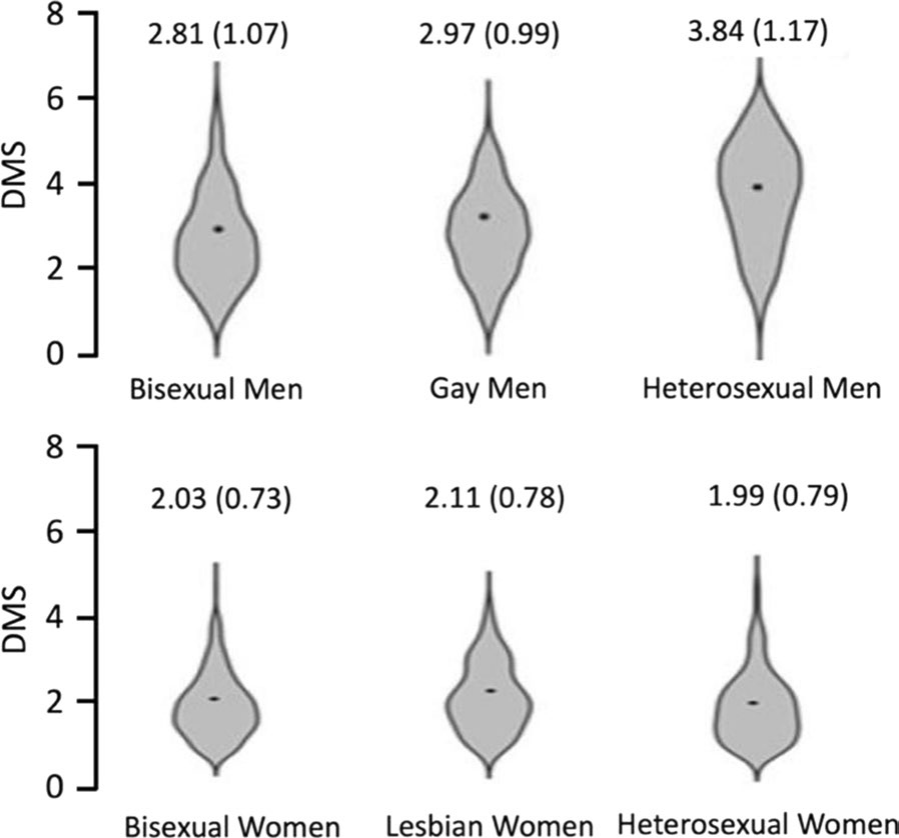
Two violin plots showing the small significant gender*sexuality interaction in Drive for Muscularity scores, such that there were no differences amongst men, but heterosexual women scores significantly higher than lesbian and bisexual women. *Note* Means and SDs (in brackets) for each group are shown in the figure

To follow up on the significant gender*sexuality interaction, we conducted further Bonferroni corrected pairwise comparisons (the Bonferroni critical *p-*value is 0.0083). Heterosexual men had statistically equivalent DMS scores to both gay men [t(515) = 1.54, *p* = 0.147, d = 0.161] and bisexual men [t(518) = 0.663, *p* = 0.508, d = 0.064]. There were no significant differences between heterosexual women and bisexual [t(451) = − 10.50, *p* < 0.001, d = − 0.921] or lesbian women [t(324) = − 9.07, *p* < 0.001, d = − 0.798]. Gay men scored higher than both heterosexual women [t(436) = 16.2, *p* < 0.001, d = 1.66] and lesbian women [t(418) = 9.26, *p* < 0.001, d = 0.938]. Due to the lack of statistical differences found in scores within genders, this is likely to reflect a main effect of gender such that men have higher DMS scores compared to women. See Fig. 4 below.

## Discussion

We used an EFA to CFA approach to explore the best-fitting model of the Eating Disorder Examination Questionnaire (EDE-Q) in a group of men and women identifying as gay/lesbian, bisexual, or heterosexual. We also conducted measurement invariance analysis to determine whether means could be compared across groups. After establishing this, we compared rates of disordered eating thoughts and behaviours across the groups alongside drive for muscularity given the increased popularity of fitspiration, which promotes a lean and muscular physique to both men and women.

As anticipated, the original EDE-Q factor structure was not a good fit for the data. Instead, the EFA indicated that a 14-item three-factor model was the best fit, which was confirmed in the CFA. The best-fitting factor structure collapsed the two Shape Concern and Weight Concern factors into one. There was a significant small effect of gender on disordered eating thoughts and behaviours, such that men scored significantly higher than women on restriction-related thoughts and behaviours. There was no difference between genders for shape and weight concern or preoccupation and eating concern. There were small to medium differences in these thoughts and behaviours based on an interaction between gender and sexual orientation. For both shape and weight concern and preoccupation and eating concern gay/lesbian participants scored higher than heterosexual participants, but bisexual participants scored similarly to heterosexual participants. For restriction-related thoughts and behaviours, gay/lesbian participants had statistically equivalent scores to heterosexual participants, whereas bisexual participants scored lower than heterosexual participants.

Findings in this study are consistent with results from previous research that also found a three-factor model, combining the Shape and Weight Concern factors, is the best fit for the EDE-Q [6, 30, 38]. Three-factor models have been supported in studies with both students and non-students [1] and in men and women [14]. Most studies supporting a three-factor model suggest that Shape and Weight Concern should be collapsed into one factor as opposed to split into two individual factors [1, 14, 58]. It should be noted that combining these factors has the potential to lose information relating to how people may feel differently about their shape compared to their weight. However, it may indicate that how people feel about their body shape and weight are related. The results from this study supported a 14-item model for men and women, unlike other results that supported a brief, 7-item model [30] or a longer 21-item model in women [58]. In comparison, a previous study indicates that a three-factor model with 16 items is a good fit for men of different sexual orientations [40].

Scalar invariance, deemed sufficient for comparing means across groups, was met for each group [7]. This suggests that our 14-item, three-factor model can be used to compare means across different gender and sexual orientation groups, which is useful when considering how disordered eating influences people in different ways. Only one previous study has examined this in sexual minority men and women, but heterosexual participants were not included in the study, meaning invariance was only established between gay/lesbian and bisexual participants [38]. The current research is the first to establish invariance between gay/lesbian, bisexual, and heterosexual participants across two genders, allowing us to compare means between these groups directly.

There were differences in rates of disordered eating thoughts and behaviours based on both gender and sexual orientation. The only difference in these scores based on gender was a small one, in that men scored significantly higher on restriction-related thoughts and behaviours than women, whereas other studies have found that women score higher than men [6]. To understand this, it is important to consider which items contributed to this factor. These questions focussed on deliberately limiting food, excluding foods, or following rules to influence shape or weight. In the original model, the Restriction factor also included items about going for long periods without food and the desire for a flat stomach. These items from the original scale fit the traditional concept of an eating disorder, and potentially one that is more influential for women in keeping with the thin-ideal and its disproportionate impact based on gender [45]. Given that these items were excluded from our 14-item model, it is perhaps less surprising that women did not score as highly as men here. The items that were included focussed on following rules and excluding certain foods to influence shape or weight. This is compatible with disordered eating behaviours more frequently associated with men, such as fasting and bulking in order to gain muscle (fasting and bulking refers to a pattern of behaviour in which a person eats very little for a certain period of time before eating a lot of specific types of food [41], related to the drive for muscularity and muscle dysmorphia that disproportionately affects men [15, 59, 60]. It might be more accurate to say that men have higher rates of muscularity-related eating behaviours, and women have higher rates of thinness-oriented eating behaviours. This should be considered further in future research.

There were also small to medium differences in rates of disordered eating thoughts and behaviours based on a gender and sexuality interaction, such that gay men scored higher than heterosexual women on all measures except for preoccupation and eating concern, for which they were statistically equivalent. This may be explained by theories suggesting that there are similarities in the sociocultural pressures on heterosexual women and gay men, as they are both attempting to attract men [16, 27, 63]. This may lead to similar shape and weight concern-related thoughts and behaviours being experienced by both groups, as they both could be said to be influenced by sociocultural pressure to conform to the thin-ideal [43, 76], but that gay men have the added pressure of being in a minority group, potentially accounting for higher scores. Interestingly, however, bisexual men did not show the same rates as gay men, which may be due to specific subcultural values or could be related to the gender of current partners and how this relates to heteronormative ideals. The caveat to this is that there were fewer items included in this scale, and we may therefore be missing some thoughts and behaviours related to shape and weight concern that apply differently to these groups. The equivalent scores between all the sexual orientation groups in women might indicate that any previously suggested protective element for lesbian women is mitigated by pressures associated with being a minority group and also the dominant pressures around body image experienced by women. Again, this does not fit with the reported experiences of bisexual women, who may be subject to different subcultural norms, this too needs to be examined in further research.

Gay men reported significantly more preoccupation and eating concern than heterosexual men. This might relate to increased rates of dieting behaviours and pressure to be both lean and muscular, alongside higher levels of body dissatisfaction, in the gay community [16]. Gay and heterosexual men scored the highest on the restrictive thoughts and behaviours, which, as mentioned above, could be related to the items that make up the subscale in this 14-item model. However, these results should be interpreted with the caveat that these differences generally had small effect sizes. Eating disorders are complex, and likely have many risk factors associated with them [68]. Although it seems there is some impact of gender and sexuality on disordered eating behaviours, these are small pieces in a much larger puzzle that includes other factors. It would be simplistic to assert that these are the sole factors driving differences in disordered eating thoughts and behaviours. However, it is useful to consider what their impact could be and how this may influence clinical presentation of ED. Based on the results from this study it is likely that both gender and sexuality have a small but important effect on the kind of thoughts and behaviours that different people experience and engage in.

These results highlight differences in disordered eating thoughts and behaviours linked to both gender and sexual orientation. Some suggest that men seem to focus on behaviours that could be linked to muscularity as well as thinness, whereas women are more concerned with staying or becoming slim [51, 52, 69]. It is not sufficient to only consider rates of behaviours; we also need to begin to uncover the motivation behind them. Women may pursue restrictive behaviours because of pressures to conform to a thin-ideal, whereas similar behaviours may be employed by men to ‘bulk up’ or become more muscular [51]. This is in line with our results that men showed higher levels of drive for muscularity than women. Both gender and sexual orientation should be considered when assessing people with disordered eating, as well as when determining how to best treat and support them [47]. Interventions that focus on thinness are likely to be less relevant or effective for heterosexual men, for example.

This study focussed on only three sexual orientations, namely heterosexual, gay, and bisexual men and women. Contemporary understanding of gender, sexuality, and relationship diversity recognise that there is a broader range of sexualities, all of which should be given consideration in future research. This could help us understand both individual experiences and the broader theory around risk factors for disordered eating. For example, asexual participants may show a different pattern of results as they are not attempting to attract a sexual partner, something that has been posited to explain the similarities between gay men and heterosexual women [16, 27, 63]. It would also be relevant to consider rates of these behaviours in pansexual participants, for whom gender may not play a part in attraction. Understanding how different sexual minority groups are affected by disordered eating and influenced by subcultural norms and values would allow us to provide better assessment and intervention for their wellbeing.

Another limitation relates to the methods of recruitment. Alongside adverts on social media, we placed an advert in Attitude magazine (both print and digital versions of the magazine). The media pack for Attitude indicates that readers tend to be gay men who are high earners in their 20 s or 30 s; it is unlikely that these readers are representative of the general population. It is not possible to confirm how many participants were recruited through the advertisement in Attitude in comparison to other recruitment methods, however taking this into account highlights the need for caution in the interpretation of these results. A portion of the participants in this study may not be representative and may show a pattern of results that are not generalisable to others. Similarly, we specifically included information about the purpose of the study in our advertisements, indeed this was essential for ethical considerations concerning informed consent. However, recruiting for a survey addressing *feelings towards the body* may mean that we obtained a self-selecting sample with body concerns as opposed to a representative sample of a larger group. However, this would be true for all the separate groups included in the study and our sample included a wide range of scores with all measures. A further limitation regarding the sample is that we used the same dataset for comparing groups as used for the scale validation (a random sample taken from across both the separate EFA and CFA samples). The EFA and CFA analysis along with tests for measurement invariance allowed confirmation of the scale equivalence to ensure we were comparing like for like across groups as recommended in best practice guidelines [70]. Thus, although this method should help protect against bias that may be present in using a scale that was not validated for use within this particular sample, future studies are needed to examine generalisability of these findings to other separate samples.

This study was also limited by only exploring these experiences in cisgender participants. Previous research indicates that being transgender might influence the disordered eating thoughts and behaviours that an individual experiences, and that validation of measures for trans people is important [9]. Given that gender was linked to the kinds of thoughts and behaviours that an individual experienced, we can assume that non-cisgender identities might have a different pattern of disordered eating. Further research should specifically consider transgender participants when exploring the role of gender as an eating disorder risk factor; whilst trans participants were included in the study they were not differentiated from cisgender participants unless they chose to declare their trans experience. Non-binary participants were also not included in this study, and it is reasonable to assume they may have different gender-related body experiences.

## Conclusion

This study explored the factor structure of the EDE-Q in gay/lesbian, bisexual, and heterosexual men and women. A three-factor 14-item model was supported, which was used to consider disordered eating thoughts and behaviours in this population. Sexual orientation and gender had small but significant effects on disordered eating thoughts and behaviours. Gay men tended to score higher for Shape and Weight Concern and Preoccupation and Eating Concern, bisexual men did not seem to have increased vulnerability and heterosexual women had statistically equivalent scores to both lesbian and bisexual women across all measures. Interestingly, Restriction and Drive for Muscularity had a distinctly different pattern, both with higher scores for men compared to women. This may reflect the changed 14-item measure that omits many of the more traditional restrictive behaviours relating to drive for thinness and thus may be more compatible with restricted diets to increase muscle mass. Results support a complex picture of factors that contribute to risk of disordered eating, for which gender and sexual orientation have different small effects depending on the construct measured. Therefore, an individual’s sexual orientation and gender should be considered amongst other risk factors in both the assessment and treatment of disordered eating. There is a relative dearth of literature exploring other sexual minorities’ experiences, such as pansexual and asexual people, indicating that research should focus on understanding these behaviours in other sexual minority populations too.

## Data Availability

The datasets used and/or analysed during the current study are available from the corresponding author on reasonable request.
